# Human Papillomavirus Vaccine Intent and Associated Factors Among the Unvaccinated University Freshmen in the Largest University Town in China

**DOI:** 10.3390/vaccines14070621

**Published:** 2026-07-15

**Authors:** Hongcen Yao, Hongmei Lu, Qi Zhu, Jinhua Shen, Xiaoya Fu, Yihan Lu

**Affiliations:** 1 Department of Immunization Planning, Songjiang District Center for Disease Control and Prevention, Shanghai 201600, China; 610252388@qq.com (H.Y.); 499346836@qq.com (Q.Z.); 1582052868@qq.com (J.S.); 2 Department of Epidemiology, Ministry of Education Key Laboratory of Public Health Safety, School of Public Health, Fudan University, Shanghai 200032, China; fuxy23@fudan.m.edu.cn

**Keywords:** China, freshman, human papillomavirus vaccine, university students, vaccine intent

## Abstract

**Background:** Human papillomavirus (HPV) vaccine has been proven a safe, necessary, and effective measure against HPV infection. However, HPV vaccine uptake remains limited among university students in China. This study investigated HPV vaccine intent among university freshmen in the largest university town in China. **Methods:** A cross-sectional online survey was conducted among unvaccinated female and male freshmen attending seven universities in Songjiang University Town, Shanghai, during 2024–2025. The questionnaire collected sociodemographic characteristics, HPV and HPV-vaccine-related knowledge, awareness, and vaccine intent. Factors associated with HPV vaccine intent were determined. **Results:** A total of 3397 valid questionnaires were collected, including female (60.02%) and male (39.98%) university freshmen. Overall, 76.12% were aware of HPV, 80.98% were aware of the HPV vaccine, and 84.31% expressed vaccine intent, with significantly higher rates among females than males (each *p* < 0.001). Socioeconomics, knowledge, awareness, sexual behavior, and HPV testing history were significantly associated with vaccine intent (each *p* < 0.05). The most common reason for no intent was perceived low risk of HPV-related diseases (40.53%). The most expected improvement measures were regulatory confirmation of vaccine safety and effectiveness (37.71%) and healthcare professionals’ recommendations (37.71%), with no gender difference (each *p* > 0.05). Notably, 4.00% refused HPV vaccination, regardless of improvement measures. Additionally, most respondents preferred financial supporting policies, regardless of gender or vaccine intent (each *p* > 0.05). **Conclusions:** University freshmen showed the disparity between high awareness/intent and low knowledge. Financial considerations may influence HPV vaccination decisions. Thus, improving knowledge, particularly among males, and providing financial support may enhance HPV vaccine intent.

## 1. Introduction

Approximately 660,000 new cases and 350,000 deaths of human papillomavirus (HPV)-associated cancers are diagnosed globally in 2022, of which the majority are cervical cancers [1]. As the third most common cancer among women aged 15–44 in China, cervical cancer causes 109,741 new cases and 59,060 deaths annually [2], accounting for 18.2% of the global burden [3]. Encouragingly, the World Health Organization (WHO) recommends HPV vaccination as an essential component of public health strategies to control HPV infection and eliminate cervical cancer, in the light of the massive amount of evidence that has proven that HPV vaccination is crucial and cost-effective in preventing HPV-related diseases, especially cervical cancer [4,5,6,7]. However, China has introduced and promoted HPV vaccines late for almost a decade. Since 2016, 2-valent, 4-valent, and 9-valent HPV vaccines of international manufacturers have been successively introduced and licensed for females aged 9–45 years in China. Since 2019, 2-valent, 9-valent, and 4-valent HPV vaccines of domestic manufacturers have also been continually licensed in China. Thus, a total of seven HPV vaccines are available in China’s mainland. However, HPV vaccine coverage in China remains low. In 2022, only 10.15% of girls aged 9–45 years had received the first dose of HPV vaccine, and substantial geographical and demographic disparities in vaccine access and uptake persisted [8]. In recent years, increasing provincial and local governments have provided subsidies for HPV vaccination programs among girls aged 9–14 in China, facilitating improving the vaccine uptake [9]. By 10 November 2025, HPV vaccination has been included in the national immunization program, providing free two-dose 2-valent HPV vaccines among girls aged 13 years old [10]. It has shed light on the HPV vaccination promotion to prevent HPV infection in the future.

Young adults aged 18–26 years old are at high risk of HPV infection [11], of which females under 21 years have the highest risk [12]. As a high-risk population featured sexually active, even unprotected, university students, they are facing an increasing risk of HPV infection, demonstrating an ideal population to receive HPV vaccines as early as possible [13]. Compared to adolescents aged 11–12 years old, university students are more likely to be open when investigated about their sexual behavior, and HPV and HPV-vaccine-related knowledge [14]. Additionally, while transiting into young adulthood, they begin to take ownership of their health and consider making medical decisions on their own, independent of parental consent [15]. Thus, we should pay attention to this young adult population for prevention and control of HPV infection, which is imperative as well as feasible. Previous studies from different regions of China have reported high HPV vaccine intent but relatively low vaccine uptake among university students [16]. It remains critical to promote mutual improvement of both rates.

In Shanghai, a total of seven universities are located in Songjiang University Town with nearly 72,000 students, which almost ranks the first in all university towns in China. Furthermore, approximately 27,000 freshmen enroll in each year. However, it remains unknown about their HPV vaccine intent and factors associated with vaccine intent. Thus, we constructed an online and anonymous survey to investigate HPV and HPV-vaccine-related knowledge among university freshmen, as well as their HPV vaccine intent and influencing factors. This study aimed to inform future interventions of HPV vaccine promotion among university freshmen in response to the WHO’s Accelerated Cervical Cancer Elimination Action Plan (2023–2030) [17].

## 2. Materials and Methods

### 2.1. Respondents and Recruitment

A cross-sectional survey was conducted among university freshmen attending 7 universities in Songjiang University Town, Shanghai, from December 2024 through February 2025. An online and anonymous questionnaire was distributed by scanning a QR code powered by www.wjx.com (Wenjuanxing platform, Changsha Ranxing Information Technology Co., Ltd., Changsha, China). Inclusion criteria were as follows: (1) students read the online informed consent form and voluntarily participated in this study; and (2) students were registered as university freshmen. Exclusion criteria were as follows: (1) students were unable to understand, speak, or read in Chinese; or (2) students had scheduled or received HPV vaccines.

Given the cross-sectional design, the sample size was estimated using the standard formula for prevalence studies: *n* = (Z_1−α/2_)^2^*p*(1 − *p*)/*d*^2^, where *n* is the required sample size, *p* is the expected prevalence, *q* = 1 − *p*, *d* is the allowable error, and α is the significance level. Assuming an expected HPV vaccine intent rate of 60.1% [18], *p* was set to 0.60, *d* = 0.1 *p*, and α = 0.05 (Z_1−α/2_ = 1.96). After accounting for an anticipated 20% non-response rate, the required sample size was determined to be 256 participants.

### 2.2. Questionnaire Design

A structured questionnaire survey was adopted to collect following information:

(1) Sociodemographic information, including gender (female and male), household registration (local and non-local), birth year, marital status (married; unmarried but had partners; and single), family size (single; 2–3 persons; 4–5 persons; and 6 persons and above), annual average household income in CNY (<100,000; 100,100–150,000; 150,100–200,000; 200,100–250,000; and >250,000), and living residence (urban and rural areas);
(2) HPV and HPV-vaccine-related knowledge, including understanding about HPV characteristics, transmission routes of HPV, risk of HPV infection, dangers of HPV-related diseases, susceptibility to HPV-related diseases, preventive measures against HPV infection, as well as understanding about benefits of HPV vaccination, vaccination schedule and other related knowledge;
(3) HPV vaccine intent and awareness, including participants’ intent to receive HPV vaccination; among those unwilling to be vaccinated, reasons for refusing HPV vaccination, including no perceived risk of HPV-related diseases, limited knowledge about HPV or HPV vaccines, concerns about vaccine safety or effectiveness, vaccine costs, and other reasons; concern about HPV infection; support for male HPV vaccination; and expected measures to improve HPV vaccine uptake;
(4) Behaviors related to HPV infection and prevention included age at sexual debut (defined as the age at first sexual intercourse) and HPV testing history (i.e., self-reported cervical HPV DNA testing used for HPV infection screening).

### 2.3. Variable Measurement

In the above questionnaire, a total of 10 questions about knowledge issues, of which 7 were about knowledge related to HPV and 3 to HPV vaccination. One point was given for each correct option; thus, the total score of knowledge was as high as 47 points. Knowledge scores were categorized into three levels according to the modified Bloom’s cut-off points, which are widely used in knowledge–attitude–practice studies [19]: low (<60%, <28.2 points), moderate (60–79%, 28.3–37.5 points), and high (≥80%, ≥37.6 points). The internal consistency of the knowledge scale was verified with a Cronbach’s α coefficient of 0.82, indicating good reliability. Furthermore, Bartlett’s spherical test was performed and the KMO statistic was 0.81 (*p* < 0.001), which could be considered a good construct validity.

Furthermore, the following items were categorized to multiple levels: concern about HPV infection (never heard of it; not concerned at all; not very concerned; never considered it; moderately concerned; and strongly concerned); support for male HPV vaccination (strongly disapprove; moderately disapprove; neutral; moderately support; and strongly support); and HPV testing (did not remember; had tested positive once; had tested positive multiple times; had tested negative; and never tested).

### 2.4. Quality Control

To avoid missing response and logically false answers, we set up compulsory response and automatic skip in the questionnaire. Additionally, questionnaires were considered invalid and excluded in the analysis, per the following quality control criteria: (1) submitted in less than 150 s; (2) repeated selection of the same option (such as repeatedly selecting the option A in all responses); or (3) selected options with regular pattern (such as repeatedly selecting the options A, B, and C in the sequence).

### 2.5. Statistical Analysis

Categorical variables were presented by number of cases and percentage (%), and continuous variables were presented by mean (standard deviation, SD). ANOVA and *t*-test were utilized to determine the difference when applicable. Chi-square test and multivariable logistic regression were used to determine the factors associated with HPV vaccine intent. Adjusted odds ratio (OR) and 95% confidence intervals (CIs) were calculated. Before fitting the multivariable logistic regression model, multicollinearity among independent variables was assessed using the variance inflation factor (VIF). Variables with VIF values < 5 were considered to have no evidence of problematic multicollinearity and were retained in the final model. The statistical significance level was fixed at a *p* value of less than 0.05. Statistical analysis was performed by using R package (version 4.3.1, R Foundation for Statistical Computing, Vienna, Austria).

### 2.6. Ethical Approval

This study was approved by the Institutional Review Board of the Fudan University School of Public Health (IRB 00002408 and FWA 00002399) under IRB #2024-10-1164. Information about the survey, including purpose and dimensions, was presented on the first page of the online questionnaire. The survey began only after participants confirmed their understanding to the information and clicked “agree” to participate in the survey independently. No monetary compensation or gifts were offered. Thus, it was considered they had provided the informed consent to the study. In addition, we did not collect any personal identifier in the questionnaire.

## 3. Results

### 3.1. Sociodemographic Characteristics of Respondents

A total of 3397 valid questionnaires were included in the final analysis, out of 3600 distributed questionnaires, yielding a response rate of 94.4%. Of the respondents, 2039 (60.02%) were female and 1358 (39.98%) were male. The average age of respondents was 19.19 ± 1.40 years old. Overall, 1876 (55.23%) were non-local students, 2943 (86.49%) came from urban areas, 3297 (97.00%) were single, and 2830 (83.31%) had never initiated sexual behavior.

### 3.2. Knowledge About HPV and HPV Vaccination

Among the respondents, 2586 (76.12%) knew about HPV, among whom the awareness rate was higher in females (1760; 86.32%) than in males (826; 60.82%) (χ^2^ = 291.456, *p* < 0.001); 2751 (80.98%) knew about HPV vaccines, with the awareness rate in females (1862; 91.42%) being significantly higher than that in males (889; 65.32%) (χ^2^ = 360.574, *p* < 0.001). Additionally, 2475 (72.86%) knew about both HPV and HPV vaccines, with higher awareness rate in females (1716; 84.21%) compared to males (55.82%) (χ^2^ = 45.864, *p* < 0.001). Moreover, 2864 respondents (759; 84.31%) had intent to take the HPV vaccine.

Moreover, the average score of knowledge about HPV and HPV vaccination was calculated to be 20.00 ± 15.63 points, with an almost doubled score in the group showing intent to accept HPV vaccination (21.67 ± 15.28 points) compared to the group showing no intent (11.06 ± 14.40 points) (*p* < 0.001; Table 1). Across the 10 knowledge items, there were significant differences between the two groups with higher scores in the group showing intent (each *p* < 0.001; Table 1).

### 3.3. HPV Vaccine Intent and Associated Factors

Among the respondents, the vaccine intent was significantly higher in female university freshmen (1897; 93.04%) than in males (967; 71.21%) (χ^2^ = 293.593, *p* < 0.001). As the majority (1897; 97.85%) of respondents were single, marital status was excluded in the analysis. Female students, non-local students, with higher annual household income, from urban areas, with higher scores of HPV and HPV-vaccine-related knowledge, with concern about HPV infection, supporting male HPV vaccination, having experienced sexual behavior, and once having tested HPV positive showed higher intent to take the HPV vaccine (each *p* < 0.05; Table 2).

Specifically, female university freshmen (OR = 2.72; 95% CI, 2.14–3.47) and non-local students (OR = 2.04; 95% CI, 1.63–2.55) were more likely to take the HPV vaccination. Additionally, higher intent was associated with higher average annual household income across the categories from below CNY 100,000 through below CNY 250,000 (each *p* < 0.05), while it was not significant in the category above CNY 250,000 (*p* = 0.214). Notably, HPV vaccine intent increased significantly along with knowledge score categories (each *p* < 0.05). In the awareness, higher concern about HPV infection (*p* < 0.05 in three categories) and higher support for male HPV vaccination (*p* < 0.05 in all categories) were significantly associated with increasing HPV vaccine intent. In the behavior, having had sexual behavior (OR = 1.43; 95% CI, 1.04–1.96) and once testing HPV positive (OR = 3.29; 95% CI, 1.29–8.42) were significantly associated with HPV vaccine intent; in contrast, tested HPV positive multiple times, tested negative, or did not test was not significant (each *p* > 0.05).

### 3.4. Reasons for No HPV Vaccine Intent and Expected Improvement Measures

**Figure 1:**
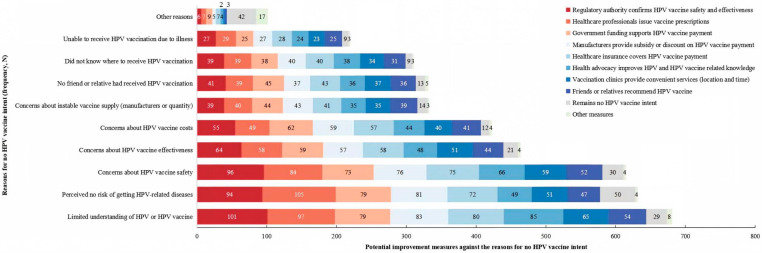
Matrix of reasons for no human papillomavirus (HPV) vaccine intent and improvement measures among university freshmen.

We further investigated the reasons and improvement measures among 533 university freshmen who were unwilling to take the HPV vaccine. The most common reason for no HPV vaccine intent was no perceived risk of HPV-related diseases (216; 40.53%), followed by limited understanding of HPV or HPV vaccines (205; 38.46%), concern about the vaccine safety (179; 33.58%) or effectiveness (120; 22.51%), and high vaccine costs (104; 19.51%) (Figure 1). Additionally, the majority of reasons were similar between males and females (each *p* > 0.05), except for two reasons, “vaccine costs” and “other reasons”, that differed between female (48; 17.14% and 31; 11.25%, respectively) and male freshmen (56; 26.06% and 47; 21.83%, respectively) (*p* = 0.022 and *p* = 0.002, respectively).

Among the respondents, the most expected improvement measures included vaccine safety and effectiveness confirmed by regulatory authority (201; 37.71%) and vaccination prescriptions issued by healthcare professionals (201; 37.71%), followed by financial support by government funding (178; 33.33%), subsidy/discount on vaccine (156; 29.27%), and medical insurance (152; 28.52%) (Figure 1). All the improvement measures were similar between female and male freshmen (each *p* > 0.05). However, 25.33% (135) of the respondents that had no HPV vaccine intent (4.00% of total respondents) remained unsatisfied with any improvement measures and refused to take the HPV vaccination, which was similar between females (67; 23.94%) and males (56; 25.83%) (χ^2^ = 0.583, *p* = 0.764).

Furthermore, 43.33% (1241) of respondents that had HPV vaccine intent and 38.84% (207) of those that had no intent considered all financially supporting policies acceptable (χ^2^ = 3.711, *p* = 0.054). However, the respondents that had intent preferred payment support for 9-valent vaccine (598; 20.88%) and for low-income families (377; 13.16%), while those that had no intent expressed no concern (172; 32.27%) and payment support for low-income families (48; 9.01%) (Table 3). Similarly, 41.16% (889) of female respondents and 43.60% (559) of male respondents considered all financially supporting policies acceptable (χ^2^ = 1.978, *p* = 0.160). It differed significantly in the preferences between males and females in the respondents with HPV vaccine intent (χ^2^ = 188.335, *p* < 0.001) or no intent (χ^2^ = 20.201, *p* < 0.001) (Table 3). In the respondents with HPV vaccine intent, females preferred payment support for 9-valent HPV vaccine (508; 26.78%), males preferred payment support for children aged 9–14 (183; 18.92%), while they both preferred payment support for low-income families (females: 251; 13.23%; males: 126; 13.03%). In contrast, the majority of respondents with no intent had no concern (females: 34; 23.94%; males: 138; 35.29%), followed by females preferring payment support for 9-valent HPV vaccine (19; 13.38%), and males preferring for low-income families (37; 9.46%).

## 4. Discussion

This study identified a high HPV vaccine intent (84.31%) among university freshmen in Shanghai, with significant gender differences favoring females. Vaccine intent was strongly associated with HPV-related knowledge, awareness, socioeconomic status, perceived risk of HPV infection, and HPV testing history. Despite high awareness and intent, overall knowledge of HPV and its vaccine remained suboptimal. In addition, most participants expressed willingness to accept financial support policies for HPV vaccination, regardless of gender or vaccine intent.

Numerous evidence has demonstrated that HPV vaccination is highly effective and cost-effective in preventing cervical cancer and HPV-related diseases. Timing of HPV vaccination initiation is an important factor in vaccine effectiveness, particularly the vaccine uptake prior to initiation of sexual behavior and potential exposure to HPV, which can provide the best protection [20]. In our study, the average age of university freshmen was 19.19 ± 1.40 years old, with 83.31% reporting no sexual behavior. Previous studies in China showed that the age of first sexual intercourse among university students was 20.14 ± 2.97 years old [21], and the HPV infection rates of females ranked first among young adults under 25 years old [22,23]. One study has shown that the first-dose (7.97%) and full-dose (4.25%) coverage rates of HPV vaccination among females aged 20–24 years ranked highest in Shanghai, followed by the coverage rate among those aged 15–19 years [24], indicating that individuals aged around 20 have relatively higher acceptance and demand for HPV vaccines. Thus, our findings suggested that timely intervention to promote HPV vaccination among the university freshmen, especially prior to the initiation of sexual behavior, would be highly significant and cost-effective.

In our study, we found considerably high awareness rate of HPV (76.12%) and the HPV vaccine (80.98%) among university freshmen. They were higher than those described before 2020 [18,25], probably attributable to the active and widespread health advocacy and education on HPV vaccination in recent years, as well as the increasing public awareness and attention to vaccination following the COVID-19 pandemic [26]. Also, the awareness rate in our study was higher than that from a study including 7 universities located in 7 geographical regions of China after 2020, with awareness rates of HPV (females, 59.9%; males, 41.8%) and HPV vaccines (females, 57.3%; males, 9.5%) [22], which might be explained by the disparity across regions. When compared internationally, a systematic review and meta-analysis encompassing global university student populations reported overall HPV awareness of 68.3% (95% CI: 56.4–79.0%) and HPV vaccine awareness of only 53.5% (95% CI: 53.0–54.1%), with actual vaccination coverage as low as 10.4% [27]. These variations may be attributable to differences in health education strategies, timing of vaccine inclusion in national immunization programs, and sociocultural contexts across countries. Regarding the participants, we included only university freshmen who had not received the HPV vaccine or made a vaccination appointment; they might largely lack related information, resulting in insufficient or incorrect knowledge about HPV or HPV vaccines [28]. Regardless of gender, the awareness rate of HPV and related diseases was lower than that of the HPV vaccine, suggesting that university freshmen might have poor health risk understanding, which results from the lack of correct information related to HPV [29]. The total score of true knowledge was low, inconsistent with a high awareness rate. This discrepancy suggests that being aware of HPV or HPV vaccination does not necessarily translate into accurate or comprehensive knowledge. Although participants were generally familiar with existing HPV vaccines, relatively lower scores were observed in domains related to HPV characteristics, vaccination recommendations, vaccination schedules, and vaccination precautions, suggesting important knowledge gaps in these areas. Notably, high awareness coupled with low accurate knowledge observed in our study is not unique to China. A global analysis revealed that only 37.3% of university students knew that HPV infection could be asymptomatic, and merely 52.2% were aware of the optimal timing for vaccination [27]. These findings indicate that current health education may have been effective in increasing public awareness but less successful in improving comprehensive HPV-related knowledge. Therefore, future educational interventions should focus on raising awareness as well as strengthening key HPV-related knowledge that supports informed vaccination decision-making. Previous studies have shown that greater awareness of HPV infection severity and susceptibility is associated with better knowledge of cervical cancer prevention [30], and the knowledge would positively impact HPV vaccine intent [31]. Therefore, future health education programs should prioritize improving comprehensive HPV-related knowledge, thereby enhancing risk perception and facilitating informed HPV vaccination decisions among university freshmen [32].

The HPV vaccine intent was 84.31% in our study, higher than other studies [16,28,33]. Furthermore, the intent was significantly higher among female freshmen (93.04%) than that among males (71.21%). Similarly, the awareness rate was significantly higher among female freshmen (86.32% of HPV and 91.42% of HPV vaccine) than that among males (60.82% and 65.32%). Females usually paid more attention to HPV vaccination issues as the initial vaccination recommendations have only focused on females for years [11]. Efforts into advocacy, education, and practice of HPV vaccination have targeted females, resulting in insufficient information and attempt for males to make a vaccination decision [34]. Worldwide, first-dose and full-dose HPV vaccination coverage among boys was merely 7% and 6% in 2023, substantially lower than the corresponding rates of 27% and 20% among girls [35]. Although high-income countries reported relatively higher coverage for boys (first-dose 57.9%), it remains lagged behind that of girls [35]. The respondents in this study were university freshmen, mostly having similar educational and social background among their peers before the college period. Our findings revealed that university freshmen from urban areas and with higher annual average household income may indicate stronger purchasing power, significantly affecting payment for HPV vaccination under the voluntary and self-paid policy [33]. Additionally, those achieving higher scores on HPV and HPV-vaccine-related knowledge, concerned about HPV infection, supporting male HPV vaccination, having had sexual behavior, and having once tested HPV positive, may gain a deeper understanding of HPV infection [36] and higher health risk perception [37]. It remains crucial to accurately identify the factors that may positively contribute to a more positive attitude to HPV vaccination and further facilitate their vaccine intent.

Moreover, we explored the reasons for no HPV vaccine intent among university freshmen. The main reasons focused on poor health risk perception, insufficient HPV and HPV-vaccine-related knowledge, concerns about the vaccine safety and effectiveness, and high vaccine costs. The lack of health education related to sexually transmitted infections including HPV infection may be an important source for poor health risk perception [38]. It has been documented that health risk perception can substantially influence HPV vaccination behavior, regardless of knowledge [31], which is consistent with our finding that 25.52% of respondents that had no vaccine intent refused to take the HPV vaccine as they did not perceive the risk of HPV-related diseases. Therefore, perceived health risk of HPV infection should be highlighted, in addition to simple health education on HPV vaccination. These barriers to vaccine intent are remarkably consistent with international findings. HPV vaccination coverage in France has persistently trailed other high-income countries due to hesitancy driven by healthcare distrust and social media-amplified safety concerns, prompting the 2018 PrevHPV multicomponent intervention combining school- and primary care-based strategies, including digital education, clinician training, and on-campus vaccination days [39]. These suggest that for university freshmen, comprehensive interventions beyond simple health education are warranted, including enhancing health risk perception, providing financial support policies, strengthening healthcare professional training, and leveraging peer education and digital tools to address safety misconceptions. We also investigated their attitudes to improvement measures. Multiple measures may turn 75% of respondents that had no vaccine intent into attitudes to take HPV vaccine. Our findings suggest that financial considerations may influence HPV vaccination decisions under the current self-paid policy. However, willingness to pay and vaccine affordability were not directly assessed in this study. Therefore, the role of cost should be interpreted with caution. The university freshmen generally supported all financially supporting policies. Furthermore, they preferred subsidy or discount on 9-valent HPV vaccines over 4-valent and 2-valent vaccines, especially among the respondents that had vaccine intent, which suggested university freshmen have significant preference for 9-valent HPV vaccines, similar to some other populations in China [40,41]. In August 2025, a 9-valent HPV vaccine of a domestic manufacturer has been licensed in China and demonstrated sustained HPV type-specific antibody levels [42]. Furthermore, multiple 9-valent vaccine candidates are in trials in China [43]. More important, since January 2025, the China National Medical Products Administration has granted expanded approval to HPV vaccines for use in males [44]. It may further reduce the vaccine prices and then possibly improve the vaccine intent and uptake.

Our study was conducted based on a large sample of university freshmen attending 7 universities in Songjiang University Town of Shanghai, covering both comprehensive and specialized types, and has produced convincing and representative findings. Thus, it provided valuable insights into factors associated with HPV vaccine intent among university freshmen. However, our study still had several limitations. First, this was a cross-sectional survey, which precluded causal inference between the identified factors and HPV vaccine intent. Reverse causality cannot be excluded; for example, students with stronger vaccine intent might have actively sought HPV-related information, resulting in higher knowledge scores rather than knowledge independently promoting vaccine intent. Second, the study focused exclusively on unvaccinated freshmen, which may have introduced selection bias and prevented direct comparisons between vaccinated and unvaccinated students, thereby limiting the generalizability of the findings to the overall university student population. Third, all participants were recruited from a single university town in Shanghai; therefore, the findings might not be generalizable to university students from rural areas, less-developed regions, or those socioeconomically diverse regions. However, Songjiang University Town enrolls students from diverse regions of China, which remains with moderate generalizability. Fourth, HPV vaccine intent rather than actual vaccine uptake was assessed. Although vaccine intent is an important predictor of subsequent behavior, the well-recognized intent–behavior gap means that intent does not necessarily translate into actual vaccination because of barriers such as vaccine availability, affordability, and changing personal circumstances. Therefore, the identified associated factors should be interpreted as determinants of vaccine intent rather than actual uptake. Finally, sexual behavior and HPV testing history were self-reported and might have been subject to recall or social desirability bias. Longitudinal multicenter studies incorporating actual vaccination outcomes are warranted to validate these findings.

## 5. Conclusions

This study determined the current HPV vaccine intent and associated factors among university freshmen and then explored the reasons for no HPV vaccine intent and potential improvement measures. Our findings highlighted the disparity between high awareness and intent of HPV vaccination and low knowledge among university freshmen. Furthermore, they preferred payment support, regardless of their gender and vaccine intent, suggesting costs might be a barrier to HPV vaccination among university freshmen. Thus, tailored health advocacy and education, especially for male students, perceived health risk of HPV-related diseases, and financially supporting policies of HPV vaccination may improve HPV vaccine intent and uptake among university freshmen.

## Figures and Tables

**Table 1:** Scores of knowledge related to HPV and HPV vaccination among university freshmen.

Knowledge Items	Total Scores	Average Scores (SD)	Scores by Vaccine Intent (SD)			
			Had HPV Vaccine Intent	Did Not Have HPV Vaccine Intent	*t* Value	*p* Value
HPV characteristics	6	2.29 (2.27)	2.47 (2.27)	1.33 (2.02)	11.659	<0.001
HPV transmission routes	5	2.20 (1.90)	2.37 (1.87)	1.30 (1.77)	12.675	<0.001
Risk of HPV infection	6	2.63 (2.31)	2.85 (2.28)	1.49 (2.12)	13.403	<0.001
Dangers of HPV-related diseases	5	2.16 (1.96)	2.33 (1.94)	1.25 (1.82)	12.386	<0.001
Susceptibility to HPV-related diseases	6	2.46 (2.22)	2.66 (2.20)	1.39 (2.02)	13.132	<0.001
Preventive measures against HPV infection	5	2.83 (2.22)	3.07 (2.16)	1.50 (2.07)	15.953	<0.001
Gender difference in HPV susceptibility	1	0.65 (0.47)	0.70 (0.45)	0.36 (0.48)	14.986	<0.001
Recommendations for HPV vaccination	6	2.25 (2.14)	2.44 (2.13)	1.22 (1.89)	13.406	<0.001
HPV vaccination schedule	1	0.56 (0.49)	0.62 (0.48)	0.28 (0.44)	15.628	<0.001
Precautions for HPV vaccination	6	1.97 (2.16)	2.16 (2.18)	0.94 (1.75)	14.153	<0.001
Total	47	20.00 (15.63)	21.67 (15.28)	11.06 (14.40)	15.466	<0.001

HPV, human papillomavirus; SD, standard deviation.

**Table 2:** Human papillomavirus (HPV) vaccine intent and associated factors among university freshmen.

Items and Categories	No. Surveyed	No. Had HPV Vaccine	Chi-Square Test		Multivariable Logistic Regression		
		Intent (%)	χ^2^	*p* Value	*p* Value	OR	95% CI
gender			293.593	<0.001			
male	1358	967 (71.21)					
female	2039	1897 (93.04)			<0.001	2.723	2.137–3.468
household registration			58.103	<0.001			
local	1521	1202 (79.03)			<0.001	2.042	1.633–2.544
non-local	1876	1662 (88.59)					
family size			2.246	0.523			
living alone	66	52 (78.79)					
2–3 persons	1756	1474 (83.94)			0.378	1.379	0.675–2.816
4–5 persons	1422	1209 (85.02)			0.332	1.427	0.695–2.930
≥6 persons	153	129 (84.31)			0.540	1.310	0.553–3.102
average annual household income (CNY)			17.301	0.002			
≤100,000	1177	953 (80.97)					
100,100–150,000	762	659 (86.48)			0.010	1.474	1.096–1.983
150,100–200,000	503	436 (86.68)			0.014	1.538	1.092–2.166
200,100–250,000	256	225 (87.89)			0.025	1.692	1.069–2.680
>250,000	699	591 (84.55)			0.214	1.210	0.896–1.635
living residence			6.157	0.013			
urban	459	369 (80.39)			0.068	1.329	0.979–1.805
rural	2983	2495 (83.64)					
concerns about HPV infection			284.606	<0.001			
never heard of it	811	540 (66.58)					
not concerned at all	311	250 (80.39)			0.398	1.197	0.788–1.819
moderately concerned	1101	1018 (92.46)			0.003	1.820	1.228–2.697
neutral	798	705 (88.35)			0.002	1.832	1.249–2.686
strongly concerned	376	351 (93.35)			0.032	1.805	1.051–3.098
knowledge of HPV and HPV vaccination			321.851	<0.001			
0 points	629	385 (61.21)					
low	1685	1479 (87.77)			0.007	1.588	1.137–2.218
moderate	464	427 (92.03)			0.006	2.023	1.220–3.353
high	619	573 (92.57)			0.002	2.134	1.321–3.446
support for male HPV vaccination			484.219	<0.001			
strongly disapprove	92	33 (35.87)					
moderately disapprove	85	53 (62.35)			0.026	2.167	1.098–4.276
neutral	822	559 (68.00)			<0.001	3.077	1.862–5.084
moderately support	785	714 (90.96)			<0.001	10.290	5.978–17.714
strongly support	1613	1505 (93.30)			<0.001	12.087	7.209–20.264
have had sexual behavior			5.755	0.016			
yes	567	497 (87.65)			0.026	1.428	1.043–1.956
no	2830	2367 (83.64)					
HPV testing			44.246	<0.001			
did not remember	696	531 (76.29)					
had tested positive once	72	66 (91.67)			0.013	3.291	1.286–8.422
had tested multiple times	31	27 (87.10)			0.632	1.329	0.415–4.256
had tested negative	202	176 (87.13)			0.682	0.897	0.532–1.511
never tested	2396	2064 (86.14)			0.283	1.147	0.893–1.473
Total	3397	2864 (84.31)					

**Table 3:** Preferences of financially supporting policies for human papillomavirus (HPV) vaccine among university freshmen.

	No. Responded (%)	Male (%)	Female (%)
Respondents with HPV vaccine intent			
All financially supporting policies are acceptable	1241 (43.33)	421 (43.54)	820 (43.23)
Government funding for children aged 9–14	362 (12.64)	183 (18.92)	179 (9.44)
Government funding for low-income families	377 (13.16)	126 (13.03)	251 (13.23)
Subsidy/discount on 2-valent HPV vaccine	61 (2.13)	30 (3.10)	31 (1.63)
Subsidy/discount on 4-valent HPV vaccine	43 (1.50)	21 (2.17)	22 (1.16)
Subsidy/discount on 9-valent HPV vaccine	598 (20.88)	90 (9.31)	508 (26.78)
Being neutral (no concern)	182 (6.35)	96 (9.93)	86 (4.53)
Respondents with no HPV vaccine intent			
All financially supporting policies are acceptable	207 (38.84)	138 (35.29)	69 (48.59)
Government funding for children aged 9–14	41 (7.69)	35 (8.95)	6 (4.23)
Government funding for low-income families	48 (9.01)	37 (9.46)	11 (7.75)
Subsidy/discount on 2-valent HPV vaccine	13 (2.44)	11 (2.81)	2 (1.41)
Subsidy/discount on 4-valent HPV vaccine	8 (1.50)	7 (1.79)	1 (0.70)
Subsidy/discount on 9-valent HPV vaccine	44 (8.26)	25 (6.39)	19 (13.38)
Being neutral (no concern)	172 (32.27)	138 (35.29)	34 (23.94)

## Data Availability

The dataset analyzed during the current study is available from the corresponding author on reasonable request.
